# 

**Published:** 1826-04

**Authors:** 


### MONTHLY CATALOGUE OF MEDICAL BOOKS.

*** *It having been hinted to us that gentlemen, sending copies of their Works, prefer having their titles given at length, it is our intention, in future, to comply with this suggestion: but it is to be observed, that no books can be entered on this List except those sent to us for the purpose;—finding, in the lists hitherto transmitted, that the names of works have frequently been given as published, which have not appeared for weeks, or even months, after.*

An Elementary Compendium of Physiology, for the Use of Students. By F. Magendie, m.d. Member of the Institute of France; Physician of the Central Chamber of Admission to the Hospitals and Municipal Charities of Paris; Member of the Philomathic and Medical Society of Emulation; of the Medical Societies of Stockholm, Copenhagen, Wilna, Philadelphia, Dublin, Edinburgh; of the Academy of Sciences of Turin, &c. Translated from the French, with copious Notes, Tables, and Illustrations, by E. Milligan, m.d. Fellow of the Royal College of Physicians, of the Society of Scottish Antiquaries; Corresponding Member of the Literary and Philosophical Societies of Manchester and Leeds, Extraordinary Member of the Royal Medical Soeiety; and Lecturer on Physiology and Therapeutics, Edinburgh. Second Edition, greatly enlarged.—Edinburgh, 1826.

A Letter, addressed to the Medical Profession, on the Encroachments on the Practice of the Surgeon-Apothecary, by a new set of Physicians. By Medico-Chirurgus.—London, 1826.

Proceedings at the Seventh Anniversary Meeting of the Hunterian Society, held on the 8th of February, 1826: with the Report, and List of Officers and Members.—London, 1826.

### NOTICE TO CORRESPONDENTS.

*Communications have been received from* Mr. Higginbottom, Dr. Venables, Dr. Lefevre, Mr. Wade, Mr. Broughton, *and* Mr. Richmond.

